# Sustained CHK2 activity, but not ATM activity, is critical to maintain a G1 arrest after DNA damage in untransformed cells

**DOI:** 10.1186/s12915-021-00965-x

**Published:** 2021-02-19

**Authors:** Iraia García-Santisteban, Alba Llopis, Lenno Krenning, Jon Vallejo-Rodríguez, Bram van den Broek, Ana M. Zubiaga, René H. Medema

**Affiliations:** 1 Department of Genetics, Physical Anthropology and Animal Physiology, University of the Basque Country (UPV/EHU), B/Sarriena s/n, 48940 Leioa, Basque Country Spain; 2 Oncode Institute, Division of Cell Biology, The Netherlands Cancer Institute, Plesmanlaan 121, 1066 CX Amsterdam, The Netherlands

**Keywords:** CHK2, ATM, G1 checkpoint, DNA damage

## Abstract

**Background:**

The G1 checkpoint is a critical regulator of genomic stability in untransformed cells, preventing cell cycle progression after DNA damage. DNA double-strand breaks (DSBs) recruit and activate ATM, a kinase which in turn activates the CHK2 kinase to establish G1 arrest. While the onset of G1 arrest is well understood, the specific role that ATM and CHK2 play in regulating G1 checkpoint maintenance remains poorly characterized.

**Results:**

Here we examine the impact of ATM and CHK2 activities on G1 checkpoint maintenance in untransformed cells after DNA damage caused by DSBs. We show that ATM becomes dispensable for G1 checkpoint maintenance as early as 1 h after DSB induction. In contrast, CHK2 kinase activity is necessary to maintain the G1 arrest, independently of ATM, ATR, and DNA-PKcs, implying that the G1 arrest is maintained in a lesion-independent manner. Sustained CHK2 activity is achieved through auto-activation and its acute inhibition enables cells to abrogate the G1-checkpoint and enter into S-phase. Accordingly, we show that CHK2 activity is lost in cells that recover from the G1 arrest, pointing to the involvement of a phosphatase with fast turnover.

**Conclusion:**

Our data indicate that G1 checkpoint maintenance relies on CHK2 and that its negative regulation is crucial for G1 checkpoint recovery after DSB induction.

**Supplementary Information:**

The online version contains supplementary material available at 10.1186/s12915-021-00965-x.

## Background

The integrity of our genome is constantly challenged by endogenous and exogenous insults that can cause damage to the DNA [1]. Double-strand breaks (DSBs) are considered the most detrimental type of lesion because they are difficult to repair and can lead to chromosomal rearrangements or losses of chromosome fragments [2]. Upon DSB induction, multiple cell cycle checkpoints ensure activation of the DNA damage response (DDR) to regulate DNA repair and cell cycle progression in a coordinated manner [3, 4]. In this sense, the cell must not only trigger a cell cycle arrest when the damage has been inflicted, but also maintain it while the lesion is being repaired and resume cell cycle progression once the repair is complete. DSBs generated by physical or chemical agents such as ionizing radiation (IR) or neocarzinostatin (NCS), activate the members of a family of phosphoinositide 3-kinase (PI3K)-related kinases (PIKKs): the ataxia telangiectasia mutated (ATM) kinase, the ATM- and Rad3-related (ATR) kinase and the DNA-dependent protein kinase catalytic subunit (DNA-PKcs) [5]. Whereas DNA-PKcs is mostly focused on promoting DSB repair, ATM and ATR also regulate cell cycle progression, ATM being the main kinase activated by DSBs [6]. ATM will phosphorylate histone H2AX in the vicinity of the damaged site to recruit repair proteins [7], as well as effector kinases to regulate cell cycle progression. The main kinase downstream of ATM is the serine/threonine-protein kinase CHK2, which is phosphorylated at threonine 68 (T68) [8], a characteristic site that can be used to measure cellular ATM activity [9]. This priming phosphorylation promotes the dimerization and autophosphorylation of CHK2 on several residues, including serine 516 (S516), which is required for the optimal activation and function of this kinase [10, 11]. The hyperphosphorylated form of CHK2 is fully active and triggers a downstream signaling cascade that activates and stabilizes p53, leading to induction of its transcriptional target p21^CIP1^, thus halting the cell cycle [12–15].

Checkpoint regulation largely depends on whether the damage was inflicted in G1 or G2 phase of the cell cycle [16]. Such dissimilar cell cycle responses might arise from fundamental differences in the regulation of the signaling pathways activated in G1 vs G2 cells upon DNA damage, although there is some overlap in the DDR signaling molecules. For instance, loss of either p53 or p21 results in complete inactivation of the G1 checkpoint, but not the G2 checkpoint [16–18], suggesting that only a G1 checkpoint-induced arrest is dependent on both proteins. Regarding the kinases that regulate checkpoint arrest after DNA damage, G2 relies mainly on ATR- and CHK1-dependent signals [19, 20], whereas G1 relies on ATM and CHK2 [9, 16]. Furthermore, life-cell imaging experiments show that untransformed cells irradiated in G2 initiate a rapid cell cycle arrest but lose their ability to recover within 1 day after IR. Conversely, cells irradiated in G1 maintain a reversible arrest for a much longer period of time, being able to recover even 3 days after DSB induction [21]. However, the mechanisms by which cells achieve a prolonged G1 arrest and the signals involved in recovery have not been elucidated.

In this work, we have employed untransformed retinal pigment epithelial (RPE-1) cells to examine G1 checkpoint regulation after DSB induction. We have discovered that while ATM phosphorylation is required for G1 checkpoint initiation, it becomes dispensable after as early as 1 h following DNA damage. Instead, we present evidence that G1 arrest maintenance relies on auto-activation of CHK2. We have found that sustained CHK2 self-activity is required for the prolonged arrest in G1 and that CHK2 phosphorylation on S516 is lost as cells recover from the damage, implying the activity of a putative phosphatase with fast turnover. Thus, phosphorylation of CHK2 on S516 would function as a critical switch controlling the timing for re-entry into the S-phase of the cell cycle.

## Results

### Induction of double-strand breaks in G1 phase activates ATM/CHK2/p53-dependent signaling responses and triggers cell cycle arrest

**Fig. 1:**
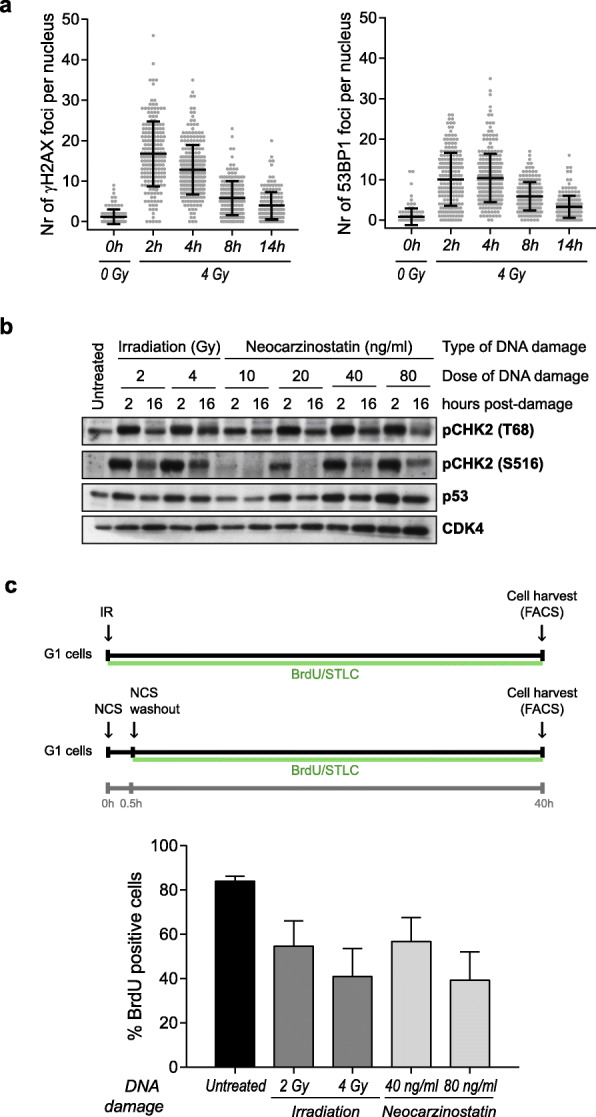
Double-strand break (DSB) induction in G1 by irradiation activates ATM/CHK2/p53-dependent signaling and triggers cell cycle arrest. **a** RPE-1 cells were fixed at the indicated times after 4 Gy IR. EdU was added at the time of IR and cells were stained for EdU using Click-iT chemistry after IF for DAPI, γH2AX, and 53BP1. The number of γH2AX and 53BP1 foci per nucleus was counted with an in-house developed macro in G1 cells, which were identified by the nuclear size (small DAPI intensity) and absence of EdU staining. **b** G1-synchronized RPE-1 cells were left untreated (0 Gy) or treated with two IR doses (2 Gy, 4 Gy), or four NCS doses (10, 20, 40, 80 ng/ml); protein was collected 2 or 16 h after DNA damage for the detection of the indicated proteins by western blot. Phosphorylation of CHK2 on T68 and S516 are indicative of active ATM and CHK2 kinases; increased p53 proteins levels are indicative of stabilized and active p53; CDK4 served as a loading control. **c** G1-synchronized RPE-1 cells were treated as in **b**; BrdU and STLC were added at the time of IR/NCS, and cells were collected by trypsinization 40 h later for flow cytometry analysis (upper panel). The percentage of BrdU-positive cells is indicative of the percentage of cells that have entered into S-phase, and decreases with IR in a dose-dependent manner. Depicted are the means and standard deviation of three independent experiments (lower panel)

In order to set up an experimental system to study DNA damage-induced G1 checkpoint regulation, we made use of untransformed, immortalized retinal pigment epithelial cells (RPE-1), as they are p53-proficient and retain a functional G1 checkpoint [21, 22]. A nearly pure G1 cell population can be obtained after growing RPE-1 cells to confluency to achieve contact inhibition, starving them for around 36 h, and re-stimulating with serum for 4 h (Additional file 1 Fig. S1a-b). Exposure of G1-synchronized RPE-1 cells to ionizing radiation (IR) triggered the formation of γH2AX and 53BP1 foci (Fig. 1a), two well-characterized markers for DNA double-strand breaks (DSBs). As expected, the high number of breaks generated during the first hours after DSB induction were rapidly repaired, with a minimum amount of breaks remaining after 14 h of DNA damage induction (Fig. 1a).

The downstream signaling cascade activated by DSBs was detected by western blot. Exposure of G1-synchronized RPE-1 cells to 2 and 4 Gy doses of IR induced the phosphorylation of CHK2 in threonine 68 (T68) and serine 516 (S516) (Fig. 1b). These phosphosites are commonly used as specific readouts for ATM and CHK2 activities, respectively, as evidenced by the disappearance of T68 or S516 phosphorylation upon pretreatment of irradiated cells with specific inhibitors for either ATM or CHK2 activities (Additional file 1 Fig. S1c-d). As shown in Fig. 1b, T68- and S516-phosphorylation were high at 2 h after the damage and could still be detected 16 h later, suggesting a rapid and persistent activation of ATM and CHK2 upon damage. In line with the wild-type p53 status of RPE-1 cells, the levels of this protein showed an initial increase, evident after 2 h post-damage, followed by a decrease at later timepoints. Similarly, treatment of G1-synchronyzed RPE-1 cells with various doses of neocarzinostatin (NCS), a well-known radiomimetic drug that also generates DSBs, led to similar dynamics of ATM, CHK2, and p53 activities (Fig. 1b).

To monitor the consequences of DSB induction on cell cycle progression, G1-synchronized RPE-1 cells were incubated with the thymidine analog BrdU to label S-phase cells, by adding the compound at the same time of IR or after the NCS pulse (Fig. 1c, upper panel). To monitor the accumulation of S-phase cells after damage, the Eg5-inhibitor STLC, which traps cells in mitosis [23], was added concomitantly with BrdU (Fig. 1c, green line). This ensures that all the BrdU-positive cells that are computed derive from a single cell cycle progression event. Forty hours after exposure to either IR or NCS, a dose-dependent decrease in BrdU incorporation was observed, indicating that both treatments hampered the progression into S-phase (Fig. 1c, lower panel).

Therefore, G1-synchronyzed RPE-1 cells activate the ATM-CHK2-p53 axis and trigger a G1 arrest upon DSB induction, making this experimental system very suitable for the study of mechanisms of G1 checkpoint regulation.

### CHK2 kinase, but not ATM kinase, maintains G1 checkpoint arrest after DNA damage

**Fig. 2:**
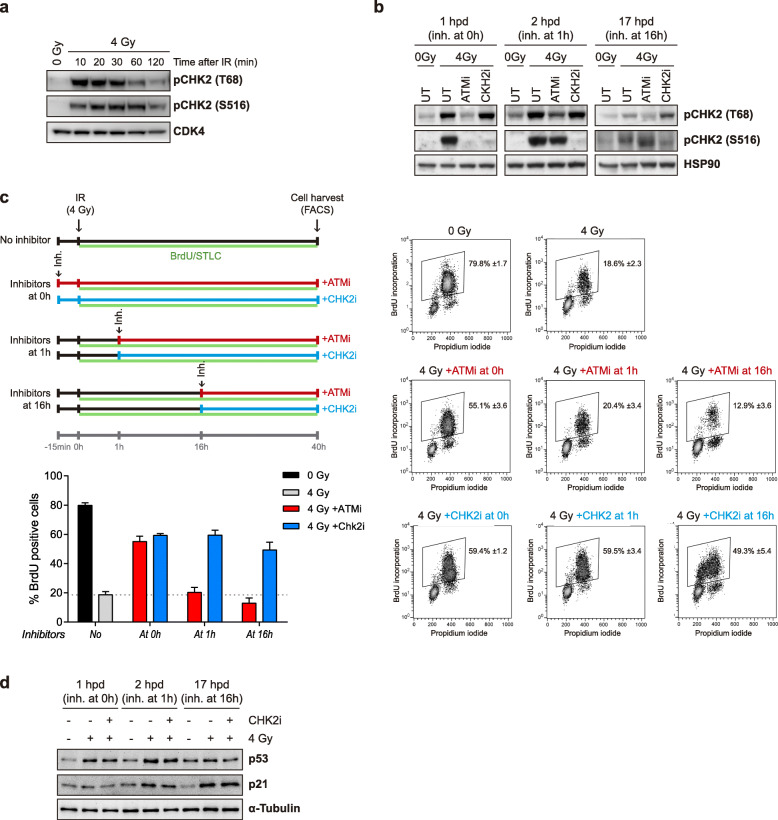
Once initiated by ATM, CHK2 kinase maintains G1 checkpoint arrest after DNA damage. **a** G1-synchronized cells were irradiated, and protein was harvested at the indicated timepoints after the damage for western blot analysis of CHK2 T68 and S516 phosphosites. **b** G1-synchronized RPE-1 cells were left unirradiated (0 Gy) or irradiated (4 Gy); irradiated cells were left untreated (UT) or treated with inhibitors for ATM (ATMi) or CHK2 (CHK2i) at the indicated times (0, 1, 16 h) and protein was harvested 1 h later (1, 2, 17 h post-damage, hpd) for western blot analysis. HSP90 served as a loading control. **c** G1-synchronized RPE-1 cells were treated as in **b**, BrdU and STLC were added at the time of IR, and cells were collected by trypsinization 40 h later for flow cytometry analysis of BrdU-positive (S-phase) cells (upper left part). In the right part, BrdU/propidium iodide plots of each experimental condition of a representative experiment are shown. In the lower left part, a bar graph represents the means and standard deviation of three independent experiments. **d** G1-synchronized RPE-1 cells were left unirradiated (0 Gy) or irradiated (4 Gy); irradiated cells were left untreated or treated with CHK2i at the indicated times (0, 1, 16 h) and protein was harvested 1 h later (1, 2, 17 hpd) for western blot analysis

Despite the well-established role of ATM and CHK2 kinases in G1 checkpoint initiation, their contribution to G1 checkpoint maintenance has not been explored in detail. Making use of the experimental setup presented in Fig. 1, we first assessed the kinetics of ATM and CHK2 activities at short times after DNA damage by monitoring the CHK2 T68 and S516 phosphosites. As shown in Fig. 2a, both ATM and CHK2 are activated as early as 10 min after irradiation, with ATM activity peaking before CHK2 activity. We next verified that ATM and CHK2 kinases function in initiating G1 checkpoint signaling by inhibiting ATM or CHK2 activity just before IR (0 h) in G1-synchronized RPE-1 cells. As shown in Fig. 2b (left panel), ATM inhibition with a specific inhibitor reduced T68 and S516 phosphorylation, indicating that it does not only abolish its own activity, but also the activation of its downstream target CHK2. By contrast, CHK2 inhibition with a specific inhibitor efficiently reduced its phosphorylation on S516 without disturbing ATM activity, as CHK2-T68 phosphorylation remained unaffected. Importantly, the pharmacological inhibitors that were used to block either ATM or CHK2 activities did not alter total CHK2 levels (Additional file 2 Fig. S2a).

Next, we evaluated the differential role of ATM and CHK2 kinases on G1-checkpoint establishment by analyzing BrdU incorporation in the presence of STLC (Fig. 2c, inhibitors at 0 h). Inhibition of either kinase around 15 min before IR (0 h) induced a near-complete override of the G1 arrest, as evidenced by a nearly 60% BrdU-positive cell population upon this treatment (Fig. 2c). A different inhibitor (obtained from Apex-Bio) led to similar results (Additional file 2 Fig. S2b). These results are consistent with the role of ATM and CHK2 on G1 checkpoint establishment. To examine the role of ATM and CHK2 kinases on G1 checkpoint maintenance, the same inhibitors were added after 1 or 16 h of IR treatment. Strikingly, inhibition of ATM at these time points no longer resulted in a reduction in CHK2 activation status, as evidenced by sustained phosphorylation on the S516 autophosphorylation site (Fig. 2b, middle and right panels). This indicates that CHK2 activity is uncoupled from its upstream activator ATM as early as 1 h after the damage is inflicted. Interestingly, this uncoupling seems to be particularly effective in G1-arrested cells, but not in G2-arrested cells, as inhibition of ATM 1 h after IR in a G2-synchronized RPE-1 cell population (Additional file 2 Fig. S2c-d) caused a clear decrease in pCHK2 S516 levels compared to the untreated control (Additional file 2 Fig. S2e).

Strikingly, BrdU incorporation analyses showed that addition of CHK2 inhibitor after 1 or 16 h following IR was unable to impose G1 arrest as cells entered S-phase very efficiently; by contrast, ATM inhibition failed to abrogate the G1 arrest (Fig. 2c). These results reveal a major role for CHK2 kinase, but not for ATM, in the maintenance of a DNA damage-induced G1 arrest. As expected, p53 was upregulated shortly after DNA damage was inflicted (1 and 2 h after IR), a raise that was followed by p21 induction. Importantly, the fast accumulation of p53 and p21 levels observed 2 h after IR was partially prevented by CHK2 inhibitor treatment (Fig. 2d).

Notably, in contrast to G1, pharmacological inhibition of CHK2 in G2-synchronized RPE-1 cells was not sufficient to induce a clear checkpoint recovery (Additional file 2 Fig. S2f), as previously reported [24]. This is likely due to the fact that end-resection is more active in G2 cells, causing co-activation of ATR and CHK1. Nonetheless, these data clearly indicate that CHK2 kinase plays a distinct role in G1 checkpoint regulation. Altogether, our findings suggest that while ATM and CHK2 activities are required for a G1 checkpoint establishment, once initiated, ATM becomes dispensable for further activities, and checkpoint maintenance mainly relies on CHK2 kinase. This raises the intriguing possibility that CHK2 auto-activation is sufficient to maintain a checkpoint-induced arrest in G1, something that could be achieved independently of the persistence of the lesion that produced its activation.

### CHK2 kinase maintains its own activity independently of ATM, ATR, and DNA-PKcs

Even though ATM is the main kinase that phosphorylates CHK2 on T68, triggering its activation, two other template-dependent kinases, namely ATR and DNA-PKcs, have been reported to phosphorylate CHK2 on the same residue in vitro [25–27]. We therefore tested the impact of ATR or DNA-PKcs kinases in the regulation of G1 checkpoint signaling in our cellular system. For that purpose, we made use of specific inhibitors for ATR (Additional file 3 Fig. S3a) and DNA-PKcs (Additional file 3 Fig. S3b).

**Fig. 3:**
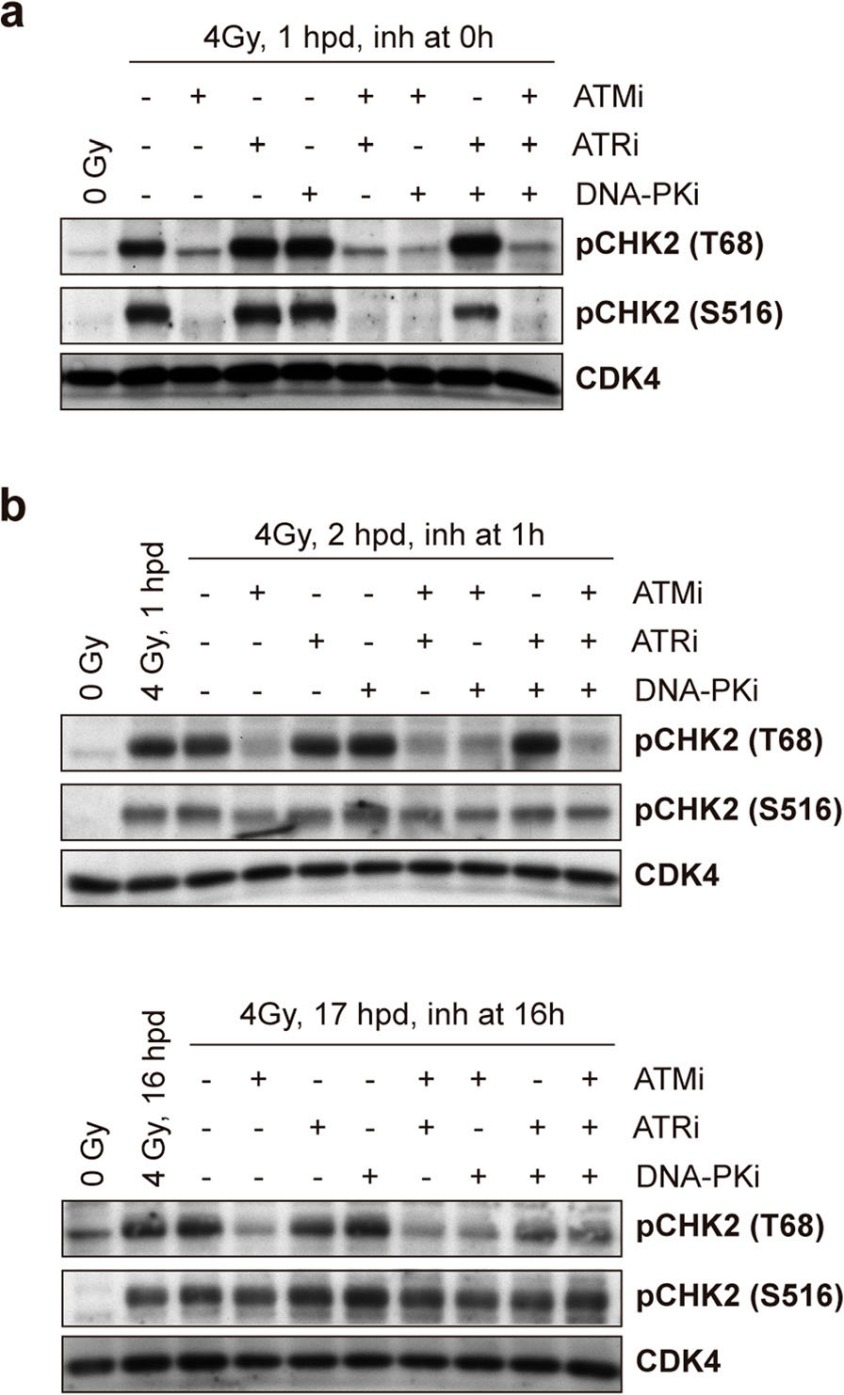
CHK2 kinase maintains its own activity independently of ATM, ATR, and DNA-PKcs after DNA damage in G1. **a** G1-synchronized cells were left unirradiated (0 Gy) or irradiated (4Gy); irradiated cells were either left untreated (−) or treated (+) with inhibitors for ATM (ATMi), ATR (ATRi), or DNA-PKcs (DNA-PKi) kinases alone or in combination at the time of IR (0 h) and protein was harvested 1 h later (1 h post-damage, hpd) for western blot analysis. **b** Cells were treated as in **a**, but inhibitors were added 1 h after IR and harvested 2hpd (upper panel) or 16 h after IR and harvested 17hpd (lower panel). CDK4 served as a loading control for all experiments

To evaluate their role in checkpoint establishment, we added inhibitors of these kinases, alone or in combination, just before IR (0 h) to G1-synchronized RPE-1 cells. As shown in Fig. 3a, only the inhibition of ATM could efficiently reduce the levels of the priming T68 phosphosite. By contrast, specific inhibition of ATR or DNA-PKcs did not prevent phosphorylation of CHK2 on T68 or on S516 in our cellular system. Furthermore, only ATM inhibition at the time of IR could fully prevent the activation of CHK2, as judged by the absence of S516 phosphorylation upon ATM inhibitor treatment. These results suggest that ATM is the major upstream kinase that triggers the activation of CHK2 in G1-synchronized RPE-1 cells.

Next, we wondered whether, once the G1 checkpoint has been initiated, any of these template-dependent kinases could phosphorylate CHK2, and keep its activity. For this purpose, we treated cells with specific inhibitors of ATM, ATR, or DNA-PKcs kinases after 1 or 16 h following IR, and checked CHK2 phosphorylation levels. We confirmed that CHK2 T68 phosphorylation, but not S516 phosphorylation, was prevented upon ATM inhibition (Fig. 3b). Furthermore, inhibition of ATR and DNA-PKcs, either alone or in combination, did not prevent phosphorylation of T68 or S516 sites on CHK2 (Fig. 3b). These data suggest that the template-dependent kinases ATM, ATR, and DNA-PKcs are dispensable for maintaining CHK2 activity in G1-arrested cells.

### CHK2 self-activation is critical to sustain the G1 arrest induced by DNA damage

**Fig. 4:**
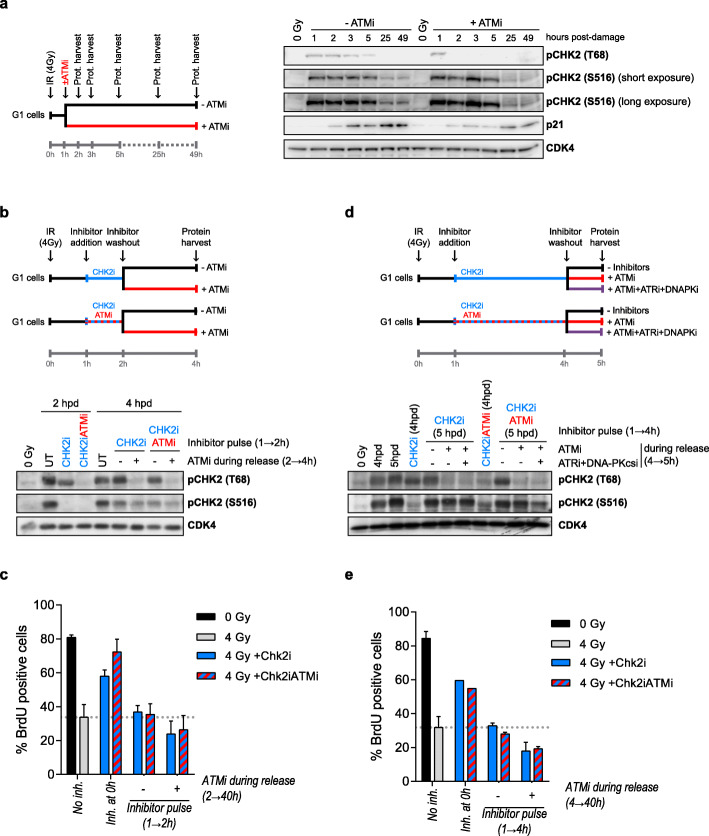
CHK2 self-activity after DNA damage enables G1 arrest establishment independently of ATM. **a** Left panel: experimental setup. G1-synchronized RPE-1 cells were irradiated with a dose of 4 Gy, and 1 h after cells were either left untreated (−ATMi) or treated with ATM inhibitor (+ATMi); protein was harvested at the indicated timepoints. Right panel: western blot analysis of protein extracts. **b** Upper panel: experimental setup. G1-synchronized RPE-1 cells were irradiated with a dose of 4 Gy, and 1 h after IR inhibitors for either CHK2 alone (CHK2i, blue line) or CHK2 and ATM (CHK2iATMi, blue and red line) were added; 1 h later (2 h timepoint) inhibitors were washed out, and cells were incubated in the absence (−ATMi, black line) or presence (+ATMi, red line) of ATM inhibitor for two additional hours (4 h timepoint). Lower panel: western blot analysis of protein extracts from 2 h and 4 h timepoints. **c** Cells treated as in **b** were cultured for 40 h with BrdU/STLC in the presence or absence of ATMi during inhibitor release and collected by trypsinization for flow cytometry analysis; a condition where inhibitors were added at the time of IR (0 h) and maintained until trypsinization was used as a control for G1 recovery. Depicted are the means and standard deviation of three independent experiments. **d** Upper panel: the experiment in **b** was repeated with an CHK2i or CHK2iATMi pulse of 3 h and 1 h release in the absence of inhibitors (−Inhibitors, black line), presence of ATM inhibitor (+ATMi, red line) or presence of ATM, ATR, and DNA-PKcs inhibitors (+ATMi+ATRi+DNAPKi, purple line). Lower panel: western blot analysis of protein extracts of 4 and 5 h post-damage (hpd). **e** Cell cycle analysis of cells treated as in **d**, and cultured in the presence of BrdU/STLC during inhibitor release

Once phosphorylated in T68, CHK2 dimerizes and autophosphorylates to become fully active [10, 11]. We hypothesized that by doing so CHK2 could continue to self-activate in an ATM-independent manner once the G1 arrest was established. We first tested this hypothesis by continuously exposing irradiated cells to ATMi and checking phosphorylated S516 CHK2 levels. As shown in Fig. 4a, treatment of irradiated cells with ATM inhibitor for an extended period after damage abolished T68 phosphorylation, but did not affect S516 phosphorylation of CHK2. Next, we irradiated G1-synchronized RPE-1 cells and 1 h later they were treated with either CHK2 inhibitor alone (CHK2i), or with a combination of CHK2 and ATM inhibitors (CHK2iATMi) (Fig. 4b, upper panel). After a pulse of 1 h, the inhibitors were washed out, and cells were incubated for two additional hours in the absence or presence of ATM inhibitor before protein harvest, in order to evaluate whether CHK2 phosphorylation in T68 might have any impact in its autophosphorylation capacity (Fig. 4b, upper panel). Strikingly, after inhibitor release, both T68 and S516 sites were readily re-phosphorylated almost to the level of the untreated cells, both in cultures treated with CHK2i as well as in cultures treated with CHK2iATMi. By contrast, the presence of ATM inhibitor in the media prevented T68 phosphorylation but not S516 phosphorylation of CHK2 (Fig. 4b, lower panel, 4hpd). The experiment was repeated at longer times after DNA damage, obtaining similar results (Additional file 4 Fig. S4). Overall, these data suggest that CHK2 might be responsible for keeping its own activity independently of ATM.

We next wondered whether re-activation of CHK2 was sufficient to arrest damaged cells in G1. To test this hypothesis, cells that were treated with inhibitors of CHK2 or CHK2 and ATM for an hour were further incubated in the presence of BrdU and STLC as previously explained. These cells showed the same level of G1 arrest as those that had not been treated with the kinase inhibitors (Fig. 4c), suggesting that re-activation of CHK2 was enough to re-establish an arrest in G1.

Additionally, we tested whether a longer pulse of CHK2 inhibitor or a combination of CHK2 and ATM inhibitors could render CHK2 inactive. The experiment was repeated by applying a 3-h inhibitor pulse, followed by 1-h release without inhibitors, or in the presence of either ATM inhibitor or a combination of ATM, +ATR, and +DNA-PKcs inhibitors. In line with the previous experiment results, CHK2 was readily re-phosphorylated in all conditions (Fig. 4d), and this re-phosphorylation was enough to re-establish a cell cycle arrest (Fig. 4e). These results underscore the importance of an active CHK2 autophosphorylation loop to achieve a robust G1 checkpoint arrest.

### Inhibition of protein synthesis affects CHK2 S516 phosphorylation levels

**Fig. 5:**
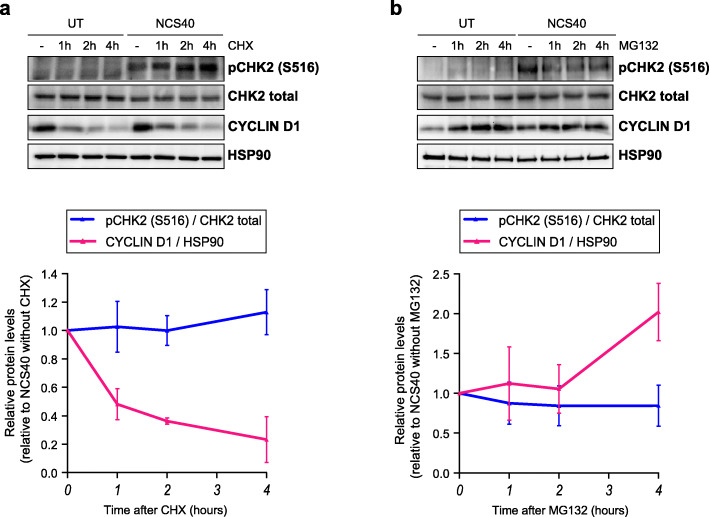
Inhibition of protein synthesis affects CHK2 S516 phosphorylation levels. **a** G1-synchronized RPE-1 cells were left untreated (UT) or treated with a dose of 40 ng/ml of neocarzinostatin (NCS40) for 30 min; around 16 h after DNA damage, cycloheximide (CHX) was added at the indicated times before protein harvest. A representative western blot is shown (upper panel) together with the quantifications of four independent experiments (lower panel). **b** RPE-1 cells were treated as in **a**, but instead of using CHX, the MG132 was added at the indicated times before protein harvest. A representative western blot is shown (upper panel) together with the quantifications of three independent experiments (lower panel). In both quantifications, pCHK2 S516 levels where relativized with total CHK2 (blue lines), while cyclin D1 levels were relativized with HSP90 (green lines); only NCS-treated conditions were quantified. Error bars indicate standard deviations

CHK2 is S516-phosphorylated and active for a relatively long time after DNA damage in G1, in part because of its ability to undergo autophosphorylation. To search for additional mechanisms that may retain CHK2 activity, we evaluated the stability of CHK2 in NCS-treated G1 RPE-1 cells in the presence of cycloheximide (CHX), an inhibitor of protein synthesis commonly used for protein stability measurements (Fig. 5a, upper panel). The level of total CHK2 did not vary upon CHX treatment, in contrast to CYCLIN D1, a protein known to have a short half-life [28]. These results suggest that CHK2 is a stable protein with a slow turnover in RPE-1 cells. Strikingly, CHX treatment caused the accumulation of pCHK2 S516 in NCS-treated cells, while it had no effect in untreated conditions. Measurement of pCHK2 S516 over total CHK2 levels in four independent experiments shows a tendency of pCHK2 S516 accumulation upon protein synthesis inhibition in DNA damaging conditions, especially when compared with the clear decrease of CYCLIN D1 levels (Fig. 5a, lower panel). The accumulation of S516 when protein synthesis inhibition is blocked is consistent with the auto-phosphorylating capacity of CHK2. Moreover, these data suggest that the removal of pCHK2 S516 phosphorylation, required to disrupt the self-activation loop in CHK2, is dependent on continued new protein synthesis.

To extend these experiments, we treated G1-synchronyzed RPE-1 cells with the proteasome inhibitor MG132 in the presence or absence of NCS. As shown in Fig. 5b, MG132 treatment did not alter total CHK2 levels, in contrast to CYCLIN D1, which accumulated after proteasome inhibition. Strikingly, pCHK2 S516 levels were slightly reduced in MG132-treated cells after DNA damage in three independent experiments. These results are consistent with our previous experiment and suggest that a CHK2 phosphatase with fast turnover could be regulating CHK2 activity, a possibility that should be further validated.

### Loss of CHK2 S516 phosphorylation is required to recover from a prolonged DNA damage-induced arrest in G1

**Fig. 6:**
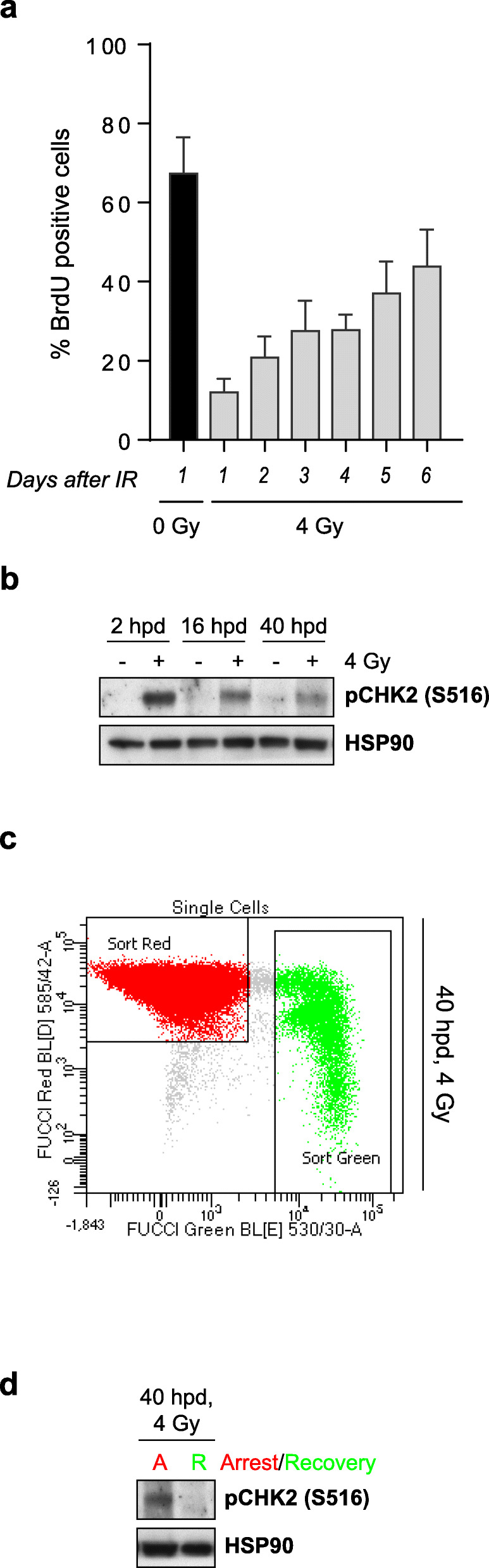
Loss of CHK2 activity is required to recover from a DNA damage-induced arrest in G1. **a** G1-synchronized RPE-1 cells were irradiated with a dose of 4 Gy; at the same time, BrdU and STLC were added and trypsinized at the indicated days after IR for flow cytometry analysis of BrdU-positive cells. Irradiated cells progressively entered into S-phase up to 6 days after IR. Depicted are the means and standard deviation of five independent experiments. **b** G1-synchronized RPE-Fucci cells were left unirradiated (−) or irradiated with a dose of 4 Gy (+); protein was harvested at the indicated hours post-damage (hpd) and analyzed by western blot. HSP90 was used as a loading control. IR induced a robust phosphorylation of CHK2 at S516 that decreased over time. **c** RPE-Fucci cells were trypsinized 40 hpd and arrested (expressing a red fluorofore) vs recovered (expressing a green fluorofore) cells were isolated using Fluorescence Activated Cell Sorting for subsequent western blot analysis. FACS plot indicates the gating windows of arrested (red) and recovered (green) cells. **d** Western blot analysis of arrested (A) vs recovered (R) cells 40 h after irradiation; pCHK2 S516 phosphorylation is lost in cells that recovered from IR

We next assessed the kinetics of recovery for cells that have been G1-arrested upon DNA damage, and analyzed the role of CHK2 S516 in this process. To this end, G1-synchronized RPE-1 cells were cultured for up to 6 days in the presence of BrdU and STLC and were harvested every 24 h. Flow cytometry analyses show that cells continued to enter into S-phase for the duration of the experiment, suggesting that G1 cells maintain the recovery competence for at least 6 days after the damage (Fig. 6a). We selected early and intermediate timepoints to assess in detail the status of CHK2 phosphorylation in cells undergoing recovery. RPE-1 cells irradiated with a dose of 4 Gy revealed that CHK2 phosphorylation on S516 was detectable for as long as 40 h after the damage (Fig. 6b), in line with the results presented in Fig. 4a. Given that at this timepoint a fraction of the cells has already recovered from the checkpoint imposed at G1, we examined S516 phosphorylation status of CHK2 using RPE-Fucci cells [29]. RPE-Fucci cells can be sorted using fluorescence-activated cell sorting (FACS); cells expressing red fluorescent mKO2-Cdt1 correspond to the G1-arrested cells, and cells expressing green fluorescent mAG-Geminin correspond to cells that recovered and had entered the cell cycle (Fig. 6c). Analysis of CHK2 phosphorylation status in RPE-Fucci cells irradiated with 4Gy revealed that while CHK2 remained phosphorylated at S516 up to 40 h in arrested cells, this phosphorylation was completely absent in recovered cycling cells (Fig. 6d), suggesting that loss of CHK2 phosphorylation at S516 is necessary to recover from a G1 arrest imposed upon DNA damage.

## Discussion

G1 checkpoint is important for cells that encounter DSBs and can be maintained for several days following the damage. However, little is known about the molecular mechanisms that regulate G1 checkpoint maintenance, partly because most cancer cell lines used in research carry genetic alterations in p53 that render G1 checkpoint non-functional [30–32]. Our aim has been to evaluate the role that ATM and CHK2, the two main kinases activated upon DSB induction in G1, play in G1 checkpoint maintenance. By using p53-proficient untransformed cells that enable sharp G1 synchronization, efficient DSB induction, reliable measurements of ATM and CHK2 activities, and easy monitoring of cell cycle progression, we present evidence that ATM and CHK2 play distinct roles in G1 checkpoint regulation.

A compelling finding presented here is that ATM activity becomes dispensable for G1 arrest maintenance very shortly after the damage has been inflicted. It has been described that inhibition of ATM at relatively long timepoints after DNA damage (16 h) did not have any impact in cell cycle regulation [22]. We have now determined that the uncoupling of ATM activity occurs as early as 1 h after damage induction and that CHK2 activity is maintained even in the continuous presence of ATM inhibitor. This implies that ATM serves mainly as a switch to initiate G1 checkpoint signaling, but subsequent maintenance of the arrest and control of the recovery largely depends on auto-activation of its direct target CHK2. Given the key role that CHK2 activity plays in G1 arrest maintenance, CHK2 S516, rather than T68, would be a more suitable marker for G1 checkpoint activity.

We have found that, unlike ATM, CHK2 activity is critical to maintain cell cycle arrest both at early and long timepoints after DSB induction in G1 cells. This is in contrast to the observation that CHK2 inhibition does not abrogate G2 checkpoint induced by UV-inflicted DNA damage [24]. Actually, in G2 phase of the cell cycle, another lesion-dependent kinase, ATR, is primarily activated, and its preferential downstream kinase is CHK1 [33, 34]. Thus, even though G1 and G2 checkpoints share a number of molecular mediators, their regulation appears to be different in each case [16, 22]. These differences in G1 vs G2 checkpoint regulation are paralleled by different kinetics of CHK2 S516 phosphorylation in each checkpoint. In agreement with previous reports, sustained ATM to CHK2 signaling helps to maintain phosphorylated CHK2 levels in G2, since ATM inhibition after initial CHK2 activation results in diminished levels of phosphorylated CHK2 [35]. In contrast, we have shown that in G1, CHK2 activity is independent of ATM, ATR, and DNA-PKcs template-dependent kinases at early and even relatively long timepoints after the damage. In fact, at the long timepoints assessed in our work (14–16 h post-damage), the majority of DNA breaks are already resolved, suggesting the existence of a “template-independent” DDR in G1. Thus, we speculate that the cell must have an independent mechanism that allows reading for the decay in breaks, opening the door for future research in the field of DNA damage response.

Once primed on T68 by ATM, CHK2 keeps its own phosphorylation and activity by different mechanisms. One of them relies on the autophosphorylation capacity of the kinase that, independently of T68 phosphorylation, enables to maintain CHK2 in a hyperphosphorylated state, as has been previously shown [36]. In our work, we demonstrate that the self-phosphorylating capacity of CHK2 is sufficient to re-establish a G1 arrest, highlighting the importance of autophosphorylation not only for sustained CHK2 activity but also for appropriate G1 checkpoint regulation. According to our data, another mechanism that might be keeping CHK2 activity is the presence of a yet unidentified phosphatase with fast turnover, as evidenced by the accumulation of CHK2 S516 upon CHX treatment.

Finally, our data clearly shows that CHK2 S516 phosphorylation is lost in cells that recover from DNA damage-induced G1 arrest, suggesting that a yet unidentified phosphatase might promote G1 checkpoint recovery. Among the phosphatases that could dephosphorylate CHK2, WIP1 has been shown to inhibit CHK2 kinase activity by dephosphorylating the T68 residue but no effect on the S516 residue has been reported [37, 38]. Furthermore, in agreement with our evidence that CHK2 activity is maintained independently of T68 phosphorylation by ATM in G1, WIP1 is only required for cell cycle re-entry in G2, but not in G1 cells [22]. Nevertheless, we still lack many pieces of the puzzle to propose a mechanism for CHK2 inactivation. Thus, further studies are required to identify the phosphatase activity responsible for CHK2 dephosphorylation and inactivation in G1 after DNA damage recovery.

## Conclusions

Taken together, our data underscore the essential role of CHK2 activity in G1 arrest maintenance and provide insights into the mechanism by which regulation of CHK2 S516 phosphorylation constitutes a critical step for G1 checkpoint decisions.

## Methods

### Cell culture, synchronization, and DSB induction

hTert-immortalized retinal pigment epithelial (RPE-1) cells were maintained in Dulbecco’s Modified Eagle’s medium/Nutrient Mixture F12 (DMEM F-12, Gibco) supplemented with fetal calf serum (FCS, 6%) and penicillin/streptomycin (1%) at 37 °C with 5% CO_2_. The RPE-Fucci cell line was constructed as previously described [22].

For G1 synchronization, RPE-1 cells grown to confluency were seeded onto appropriate wells/plates, and were left until they attach for at least 4 h. Subsequently, cells were washed three times with phosphate-buffered saline (PBS), and fresh serum-free medium was added before incubation for additional 36 h for G0 establishment. In order to induce re-entry into the cell cycle, medium was replaced with 6% FCS-containing growth medium, and cells were incubated for 4 h, a timepoint where the majority of the cells are in G1 phase, before the G1/S checkpoint (Additional file 1 Fig. S1a-b). For G2 synchronization, RPE-1 cells were seeded, left until they attach, and incubated with thymidine for 24 h followed by a 7-h release, a timepoint where the majority of the cells are in G2 phase, before the G2/M checkpoint (Additional file 2 Fig. S1c-d).

Synchronized cells were either γ-irradiated (2–4 Gy) using a Gammacell Extractor (Best Theratronics) with a cesium-137 source, or left unirradiated; alternatively, cells were treated with a pulse of neocarzinostatin for 30 min and washed two times with PBS (*Note*: the irradiation/NCS treatment time is considered as the 0 h timepoint in all the experiments, as shown in Fig. 1c).

### Chemicals and antibodies

The chemicals used for this study were the following: ataxia telangiectasia mutated inhibitor (ATMi; KU55933, 10 μM; Sigma-Aldrich), ATM- and Rad3-related inhibitor (ATRi, VE-821, 5 μM; Selleckchem), DNA-PKcs inhibitor (NU7441, 1 μM; Cayman Chemicals), CHK2 inhibitor II (CHK2i; 10 μM; Sigma-Aldrich), thymidine (2.5 mM, Sigma-Aldrich), neocarzinostatin (NCS, 40–80 ng/ml; Sigma-Aldrich), 5-bromo-2′-deoxyuridine (BrdU, 10 μM; Sigma-Aldrich), (+)-S-Trityl-l-cysteine (STLC, 20 μM; Sigma-Aldrich), 5′-ethynyl-2′-deoxyuridine (EdU, 20 μM; Thermo Fisher Scientific), propidium iodide (PI, Sigma-Aldrich), RNAse A (Sigma-Aldrich), cycloheximide (CHX, 10 μg/ml; Sigma-Aldrich), and MG132 (5 μM; Sigma-Aldrich).

The following primary antibodies were used: anti-pS139-H2AX (#05–636, Millipore), anti-53BP1 (sc-22760, Santa Cruz), anti-phospho-CHK2 T68 (2661S; Cell Signaling), anti-phospho-CHK2 S516 (2669S; Cell Signaling), anti-total-CHK2 (sc-9064; Santa Cruz); anti-p53 (sc-126; Santa Cruz), anti-p21 (sc-397, Santa Cruz), anti-CYCLIN D1 (sc-718; Santa Cruz), anti-HSP90 (sc7947 & sc-13119; Santa Cruz), anti-CDK4 (C-22; Santa Cruz), anti-BrdU (ICR1; Abcam), anti-pMPM2 (05-368; Millipore). The following secondary antibodies were used: peroxidase-conjugated goat anti-rabbit and goat anti-mouse antibodies (DAKO & Jackson laboratories), and Alexa Fluor (AF)-conjugated secondary antibodies (Molecular Probes).

### Immunofluorescence

Cells grown onto coverslips were fixed at the indicated timepoints after IR in PBS-buffered 3.7% formaldehyde (Sigma-Aldrich), permeabilized with 0.5% Triton-X100 (Sigma-Aldrich) and blocked 3% BSA in PBS before primary and fluorescence-conjugated secondary antibody incubation for immunofluorescence. Incorporated EdU was visualized using click chemistry [100 mM Tris (pH 8.5), 100 mM ascorbic acid, 1 mM CuSO_4_, 1:4000 Alexa Fluor 488-azide (Thermo Fisher Scientific)] as previously described [22]. Nuclei were counterstained with 4′,6-diamidino-2-phenylindole (DAPI; Sigma-Aldrich). Coverslips were mounted in Vectashield (Vector Laboratories). Images were taken using a Leica SP5 confocal microscope, and the amount of IR-induced foci were counted by a custom-written ImageJ macro that enabled automatic and objective analysis of the foci as previously described [39]. The updated version of the macro is available at the GitHub repository (https://github.com/bvandenbroek/nuclear_foci). G1/S cells were differentiated from G2 cells based on total DAPI intensity, and S-phase cells were identified by EdU-positivity. Thus, G1 cells were those that showed smaller DAPI intensity and were negative for EdU.

### Western blot

Cells were harvested and lysed using Laemmli buffer (120 mM Tris (pH 6.8), 4% SDS, 20% glycerol). Protein concentration was determined by Lowry method. Equal amounts of proteins were separated by SDS-PAGE, transferred to nitrocellulose membranes, and blocked in 3% bovine serum albumin (BSA, Sigma-Aldrich) in TTBS. Membranes were stained with the indicated antibodies and visualized using an enhanced chemiluminescence detection system. Band intensities were quantified with ImageJ. Uncropped western blots are shown in Additional file 5 Fig. S5.

### Flow cytometry

For flow cytometry analysis of G1 recovery, BrdU and STLC drugs were added just after irradiation (0 h timepoint). BrdU is a thymidine analog that labels S-phase cells, and STLC is a mitotic trap that prevents cells from entering into the next cell cycle round. Both BrdU and STLC were kept in culture from the time of IR until cell harvest. At the indicated timepoints, cells were trypsinized and fixed in − 20 °C 70% ethanol. Following treatment with 2 M hydrochloric acid and 0.1% Triton-X100, cells were stained with anti-BrdU and Alexa Fluor 647-coupled goat anti-rat, and anti-pMPM2 and Alexa Fluor 488-coupled goat anti-mouse antibodies, followed by propidium iodide and RNase A incubation. Data from at least 10,000 cells were collected on a Becton Dickinson FACSCalibur flow cytometer, and the percentage of BrdU-positive cells was analyzed by CellQuest software (Becton Dickinson). For analysis of G2 recovery, the same procedure was repeated in G2-synchronized cells, and the percentage of BrdU-negative and pMPM2-positive (mitotic cell readout) cells was analyzed.

### Fluorescence-activated cell sorting (FACS)

G1-synchronized RPE-Fucci cells were either irradiated or left unirradiated, and subsequently grown in the presence of STLC. At the indicated timepoints, cells were trypsinized and resuspended in growth medium diluted in PBS (1:3 dilution) for sorting using a Becton Dickinson FacsAria Sorter. G1-arrested (red) vs recovered (green) cells were sorted based on Kusabira-Orange (KO) or Azami-Green positive signal. Cells were pelleted by centrifugation and lysed in Laemmli buffer for protein isolation and subsequent western blot analysis as described above.

## Supplementary Information

**Additional file 1: Fig. S1.** G1 cell cycle synchronization and controls of ATM and CHK2 inhibitors. a RPE-1 cells were grown to confluency until they achieved contact inhibition; confluent cells were seeded, allowed to attach for 4 hours, washed three times with PBS and left cultures in starvation media for at least 36 hours for G0 establishment. Cells were re-stimulated with serum-containing medium for 4 hours to obtain a G1-enriched population. b G1 cells were collected by trypsinization, stained with propidium iodide (PI) and analyzed by flow cytometry. PI profile shows that 90% of RPE-1 cells are in G1 phase after the synchronization protocol. c RPE-1 cells irradiated with a dose of 4 Gy were left untreated or pre-treated with ATM inhibitor (ATMi) before IR; cells were harvested 1 hour after IR. Cells treated with ATMi show lower pCHK2 T68 levels, similar to the non-irradiated counterparts (0 Gy), indicating that pCHK2 T68 phosphosite is a good readout for ATM activity. d RPE-1 cells irradiated with a dose of 4 Gy were left untreated or pre-treated with CHK2 inhibitor (CHK2i) before IR; cells were harvested 1 hour after IR. Cells treated with CHK2i show lower pCHK2 S516 levels, similar to the non-irradiated counterparts (0 Gy), indicating that pCHK2 S516 phosphosite is a good readout for CHK2 activity.

**Additional file 2: Fig. S2.** CHK2 activity is required to maintain cell cycle arrest after DSB induction in G1, but not in G2. a RPE-1 cells irradiated with 4 Gy and treated with specific inhibitors for ATM and CHK2 show similar levels of total CHK2 protein. b Left panel: G1-synchronized RPE-1 cells were irradiated (4 Gy) and treated with the indicated doses of either CHK2i-II or CHK2i from Apex-Bio for one hour; protein extracts were analyzed by western blot. Right panel: G1 cells treated as in b with 10 µM and 0.5 µM concentrations of CHK2i-II or CHK2i-Apex-Bio, respectively, were further incubated with BrdU/STLC, and BrdU incorporation was analyzed by flow cytometry. c G2-synchronization protocol. Asynchronous RPE-1 cells were seeded, allowed to attach for approximately 24 hours, and blocked in the G1/S boundary with thymidine for 24 hours; cells were released for 7 hours to obtain a G2-enriched population. d PI profile of G2 cells shows that 90% of RPE-1 cells are in G2 phase after the synchronization protocol. e G2-synchronized RPE-1 cells were left unirradiated (0 Gy) or irradiated (4 Gy); irradiated cells were left untreated (UT) or treated with inhibitors for ATM (ATMi) or CHK2 (CHK2i) at the indicated times (0 or 1 hour after IR) and protein was harvested at 2 hours post-damage timepoint for western blot analysis. CDK4 served as a loading control. f G2-synchronized RPE-1 cells were treated with CHK2i or ATRi (positive control); BrdU and STLC were added at the time of IR, and cells were collected by trypsinization for flow cytometry analysis of mitotic cells that were in G2 at the time of IR (BrdU-negative/MPM2-positive). Statistical analysis was carried out using one-way ANOVA (n.s.: non-significant; **p<0.01).

**Additional file 3: Fig. S3.** Controls for ATR and DNA-PKcs inhibitors used in the present work. a RPE-1 cells were treated with hydroxyurea (HU) to induce ATR activation, or pre-treated with ATR inhibitor before HU treatment. Protein was harvested, and ATR activation status was checked by western blot using pCHK1 S345 phopshosite as a readout. CDK4 served as a loading control. ATRi effectively prevented HU-induced pCHK2 phosphorylation. b G1-synchronized RPE-1 cells grown onto coverslips were irradiated (4 Gy), and fixed for γH2AX and DAPI staining at the indicated timepoints; one sample was pretreated with DNA-PKcs inhibitor. Treatment with DNA-PKcsi prevented the foci resolution observed at 40 hours post-damage.

**Additional file 4: Fig. S4.** CHK2 self-activity at long timepoints after DNA damage enables G1 arrest establishment. Upper panel: experimental setup. G1-synchronized RPE-1 cells were irradiated with a dose of 4 Gy, and 16h after IR inhibitors for either CHK2 alone (CHK2i, blue line) or CHK2 and ATM (blue and red line) were added; one hour later (17h timepoint) inhibitors were washed out, and cells were incubated in the absence (-ATMi, black line) or presence (+ATMi, red line) of ATM inhibitor for two additional hours (19 h timepoint). Lower panel. western blot analysis of protein extracts from 17h and 19h timepoints; left panel corresponds to cells treated with CHK2i alone, and right panel to cells treated with a combination of CHK2i and ATMi.

**Additional file 5: Fig. S5.** Uncropped western blots.

## Data Availability

All data generated or analyzed during this study are included in this published article and its supplementary information files.

## References

[1] Hakem R (2008). DNA-damage repair; the good, the bad, and the ugly. EMBO J.

[2] Hoeijmakers JHJ (2009). DNA damage, aging, and cancer. N Engl J Med.

[3] Zhou BBS, Elledge SJ (2000). The DNA damage response: putting checkpoints in perspective. Nature..

[4] Langerak P, Russell P (2011). Regulatory networks integrating cell cycle control with DNA damage checkpoints and double-strand break repair. Philosophical Trans Royal Soc B: Biological Sci.

[5] Blackford AN, Jackson SP (2017). ATM, ATR, and DNA-PK: the trinity at the heart of the DNA damage response. Mol Cell.

[6] Shiloh Y, Ziv Y (2013). The ATM protein kinase: regulating the cellular response to genotoxic stress, and more. Nat Rev Mol Cell Biol.

[7] Burma S, Chen BP, Murphy M, Kurimasa A, Chen DJ (2001). ATM phosphorylates histone H2AX in response to DNA double-strand breaks. J Biol Chem.

[8] Ahn JY, Schwarz JK, Piwnica-Worms H, Canman CE (2000). Threonine 68 phosphorylation by ataxia telangiectasia mutated is required for efficient activation of Chk2 in response to ionizing radiation. Cancer Res.

[9] Rainey MD, Charlton ME, Stanton RV, Kastan MB (2008). Transient inhibition of ATM kinase is sufficient to enhance cellular sensitivity to ionizing radiation. Cancer Res.

[10] Lee CH, Chung JH (2001). The hCds1 (Chk2)-FHA domain is essential for a chain of phosphorylation events on hCds1 that is induced by ionizing radiation. J Biol Chem.

[11] Schwarz JK, Lovly CM, Piwnica-Worms H (2003). Regulation of the Chk2 protein kinase by oligomerization-mediated cis- and trans-phosphorylation. Mol Cancer Res.

[12] Chehab NH, Malikzay A, Appel M, Halazonetis TD (2000). Chk2/hCds1 functions as a DNA damage checkpoint in G1 by stabilizing p53. Genes Dev.

[13] Shieh SY, Ahn J, Tamai K, Taya Y, Prives C (2000). The human homologs of checkpoint kinases Chk1 and Cds1 (Chk2) phosphorylate, p53 at multiple DNA damage-inducible sites. Genes Dev.

[14] Chehab NH, Malikzay A, Stavridi ES, Halazonetis TD (1999). Phosphorylation of Ser-20 mediates stabilization of human p53 in response to DNA damage. Proc Natl Acad Sci U S A.

[15] Harper JW, Elledge SJ, Keyomarsi K, Dynlacht B, Tsai LH, Zhang P (1995). Inhibition of cyclin-dependent kinases by p21. Mol Biol Cell.

[16] Shaltiel IA, Krenning L, Bruinsma W, Medema RH (2015). The same, only different - DNA damage checkpoints and their reversal throughout the cell cycle. J Cell Sci.

[17] Kastan MB, Onyekwere O, Sidransky D, Vogelstein B, Craig RW (1991). Participation of p53 protein in the cellular response to DNA damage. Cancer Res.

[18] Deng C, Zhang P, Wade Harper J, Elledge SJ, Leder P (1995). Mice lacking p21 CIP1/WAF1 undergo normal development, but are defective in G1 checkpoint control. Cell..

[19] Liu Q, Guntuku S, Cui XS, Matsuoka S, Cortez D, Tamai K (2000). Chk1 is an essential kinase that is regulated by Atr and required for the G2/M DNA damage checkpoint. Genes Dev.

[20] Brown EJ, Baltimore D (2003). Essential and dispensable roles of ATR in cell cycle arrest and genome maintenance. Genes Dev.

[21] Krenning L, Feringa FM, Shaltiel IA, VandenBerg J, Medema RH (2014). Transient activation of p53 in G2 phase is sufficient to induce senescence. Mol Cell.

[22] Shaltiel IA, Aprelia M, Saurin AT, Chowdhury D, Kops GJ, Voest EE (2014). Distinct phosphatases antagonize the p53 response in different phases of the cell cycle. Proc Natl Acad Sci U S A.

[23] Kaan HYK, Weiss J, Menger D, Ulaganathan V, Tkocz K, Laggner C (2011). Structure-activity relationship and multidrug resistance study of new S-trityl-L-cysteine derivatives as inhibitors of Eg5. J Med Chem.

[24] Warmerdam DO, Brinkman EK, Marteijn JA, Medema RH, Kanaar R, Smits VAJ (2013). UV-induced G2 checkpoint depends on p38 MAPK and minimal activation of ATR-Chk1 pathway. J Cell Sci.

[25] Matsuoka S, Rotman G, Ogawa A, Shiloh Y, Tamai K, Elledge SJ (2000). Ataxia telangiectasia-mutated phosphorylates Chk2 in vivo and in vitro. Proc Natl Acad Sci U S A.

[26] Wang XQ, Redpath JL, Fan ST, Stanbridge EJ (2006). ATR dependent activation of Chk2. J Cell Physiol.

[27] Li J, Stern DF (2005). Regulation of CHK2 by DNA-dependent protein kinase. J Biol Chem.

[28] Masclef L, Dehennaut V, Mortuaire M, Schulz C, Leturcq M, Lefebvre T, et al. Cyclin D1 stability is partly controlled by O-GlcNAcylation. Front Endocrinol (Lausanne). 2019;10(106):12. https://www.frontiersin.org/articles/10.3389/fendo.2019.00106/full. 10.3389/fendo.2019.00106 PMC6395391 30853938

[29] Sakaue-Sawano A, Kurokawa H, Morimura T, Hanyu A, Hama H, Osawa H (2008). Visualizing spatiotemporal dynamics of multicellular cell-cycle progression. Cell..

[30] Sophie Mokas JRM, Cristina Garreau M-J, Fournier ´E, Robert F, Arya P, Kaufman RJ, et al. Uncoupling stress granule assembly and translation initiation inhibition. Mol Biol Cell 2009;20:2673–2683. 10.1091/mbc.E08-10-1061 PMC2688547 19369421

[31] Chen Z, Xiao Z, Chen J, Ng SC, Sowin T, Sham H (2003). Human Chk1 expression is dispensable for somatic cell death and critical for sustaining G2 DNA damage checkpoint. Mol Cancer Ther.

[32] Menzel T, Näsignhse-Kumpf V, Kousholt AN, Klein DK, Lund-Andersen C, Lees M (2011). A genetic screen identifies BRCA2 and PALB2 as key regulators of G2 checkpoint maintenance. EMBO Rep.

[33] Hekmat-Nejad M, You Z, Yee M Ching, Newport JW, Cimprich KA. Xenopus ATR is a replication-dependent chromatin-binding protein required for the DNA replication checkpoint. Curr Biol 2000;10:1565–1573. 10.1016/s0960-9822(00)00855-1 11137007

[34] Zhao H, Piwnica-Worms H (2001). ATR-mediated checkpoint pathways regulate phosphorylation and activation of human Chk1. Mol Cell Biol.

[35] Shibata A, Barton O, Noon AT, Dahm K, Deckbar D, Goodarzi AA (2010). Role of ATM and the damage response mediator proteins 53BP1 and MDC1 in the maintenance of G2/M checkpoint arrest. Mol Cell Biol.

[36] Ahn J, Prives C (2002). Checkpoint kinase 2 (Chk2) monomers or dimers phosphorylate Cdc25C after DNA damage regardless of threonine 68 phosphorylation. J Biol Chem.

[37] Oliva-Trastoy M, Berthonaud V, Chevalier A, Ducrot C, Marsolier-Kergoat MC, Mann C (2007). The Wip1 phosphatase (PPM1D) antagonizes activation of the Chk2 tumour suppressor kinase. Oncogene..

[38] Fujimoto H, Onishi N, Kato N, Takekawa M, Xu XZ, Kosugi A (2006). Regulation of the antioncogenic Chk2 kinase by the oncogenic Wip1 phosphatase. Cell Death Differ.

[39] Warmerdam DO, Alonso-de Vega I, Wiegant WW, van den Broek B, Rother MB, Wolthuis RM, et al. PHF6 promotes non-homologous end joining and G2 checkpoint recovery. EMBO Rep. 2020;21:e48460. https://www.embopress.org/doi/full/10.15252/embr.201948460. 10.15252/embr.201948460 PMC6944915 31782600

