# Supplementary figures and images for: Sustained CHK2 activity, but not ATM activity, is critical to maintain a G1 arrest after DNA damage in untransformed cells
Source: BMC Biol. 2021 Feb 19;19:35. doi: 10.1186/s12915-021-00965-x (PMC7896382; doi:10.1186/s12915-021-00965-x)

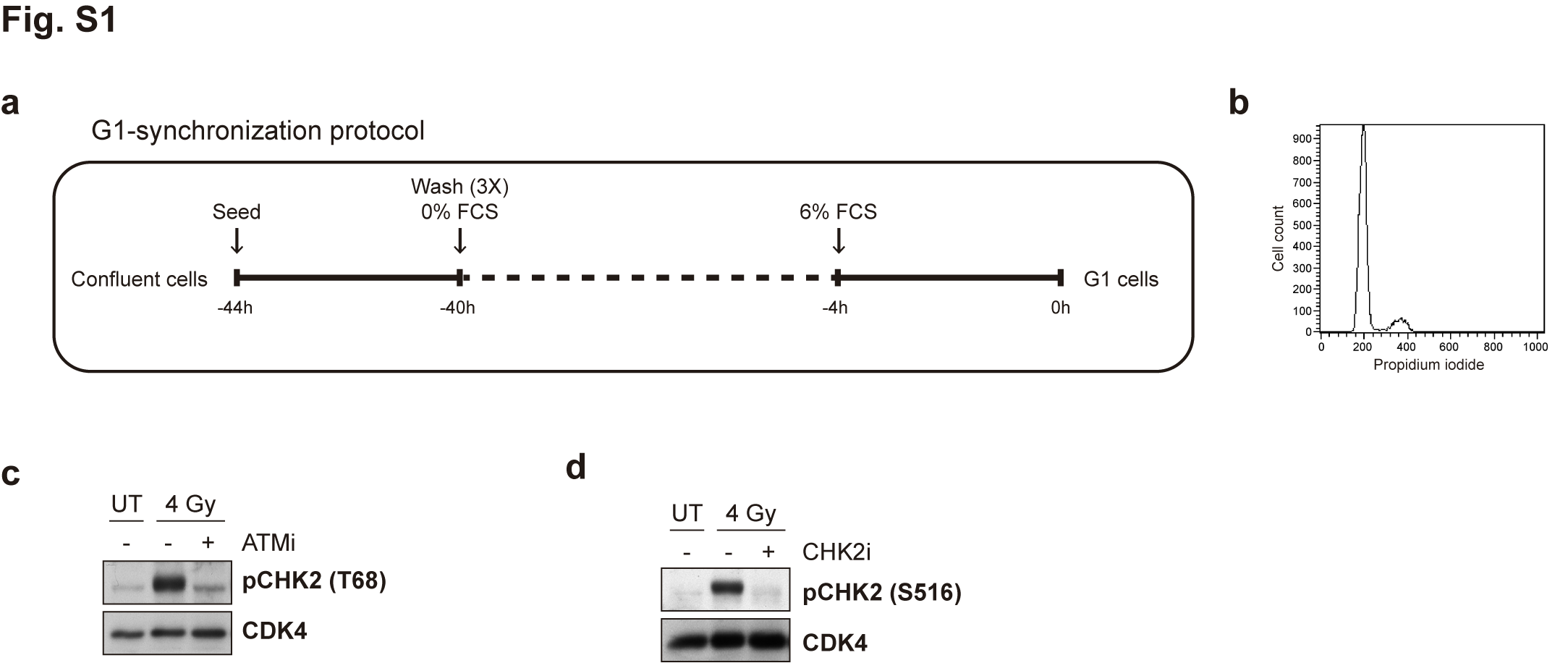

Supplement: Supplementary file 1 — Additional file 1: Fig. S1. G1 cell cycle synchronization and controls of ATM and CHK2 inhibitors. a RPE-1 cells were grown to confluency until they achieved contact inhibition; confluent cells were seeded, allowed to attach for 4 hours, washed three times with PBS and left cultures in starvation media for at least 36 hours for G0 establishment. Cells were re-stimulated with serum-containing medium for 4 hours to obtain a G1-enriched population. b G1 cells were collected by trypsinization, stained with propidium iodide (PI) and analyzed by flow cytometry. PI profile shows that 90% of RPE-1 cells are in G1 phase after the synchronization protocol. c RPE-1 cells irradiated with a dose of 4 Gy were left untreated or pre-treated with ATM inhibitor (ATMi) before IR; cells were harvested 1 hour after IR. Cells treated with ATMi show lower pCHK2 T68 levels, similar to the non-irradiated counterparts (0 Gy), indicating that pCHK2 T68 phosphosite is a good readout for ATM activity. d RPE-1 cells irradiated with a dose of 4 Gy were left untreated or pre-treated with CHK2 inhibitor (CHK2i) before IR; cells were harvested 1 hour after IR. Cells treated with CHK2i show lower pCHK2 S516 levels, similar to the non-irradiated counterparts (0 Gy), indicating that pCHK2 S516 phosphosite is a good readout for CHK2 activity. [file 12915_2021_965_MOESM1_ESM.tif]

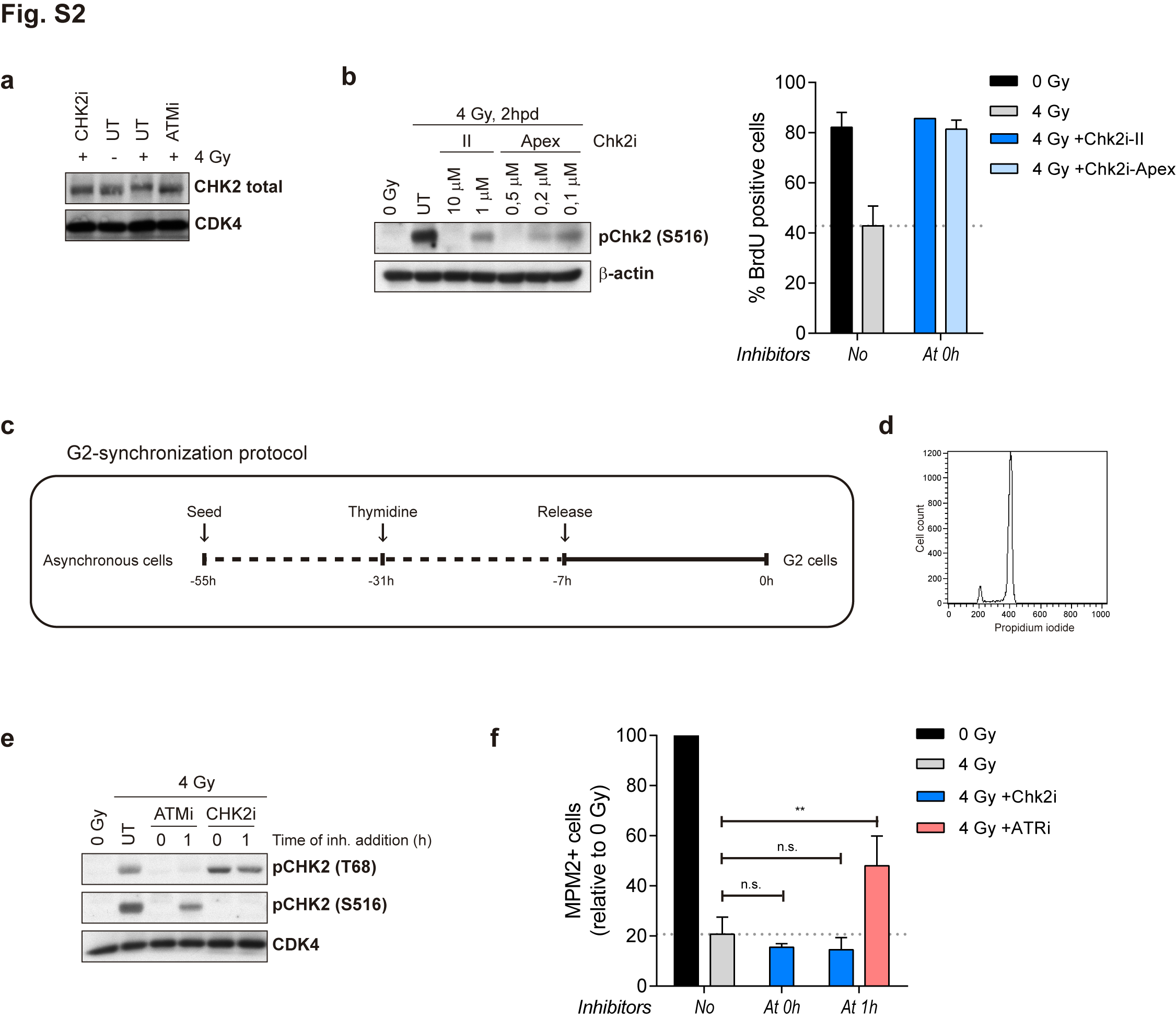

Supplement: Supplementary file 2 — Additional file 2: Fig. S2. CHK2 activity is required to maintain cell cycle arrest after DSB induction in G1, but not in G2. a RPE-1 cells irradiated with 4 Gy and treated with specific inhibitors for ATM and CHK2 show similar levels of total CHK2 protein. b Left panel: G1-synchronized RPE-1 cells were irradiated (4 Gy) and treated with the indicated doses of either CHK2i-II or CHK2i from Apex-Bio for one hour; protein extracts were analyzed by western blot. Right panel: G1 cells treated as in b with 10 µM and 0.5 µM concentrations of CHK2i-II or CHK2i-Apex-Bio, respectively, were further incubated with BrdU/STLC, and BrdU incorporation was analyzed by flow cytometry. c G2-synchronization protocol. Asynchronous RPE-1 cells were seeded, allowed to attach for approximately 24 hours, and blocked in the G1/S boundary with thymidine for 24 hours; cells were released for 7 hours to obtain a G2-enriched population. d PI profile of G2 cells shows that 90% of RPE-1 cells are in G2 phase after the synchronization protocol. e G2-synchronized RPE-1 cells were left unirradiated (0 Gy) or irradiated (4 Gy); irradiated cells were left untreated (UT) or treated with inhibitors for ATM (ATMi) or CHK2 (CHK2i) at the indicated times (0 or 1 hour after IR) and protein was harvested at 2 hours post-damage timepoint for western blot analysis. CDK4 served as a loading control. f G2-synchronized RPE-1 cells were treated with CHK2i or ATRi (positive control); BrdU and STLC were added at the time of IR, and cells were collected by trypsinization for flow cytometry analysis of mitotic cells that were in G2 at the time of IR (BrdU-negative/MPM2-positive). Statistical analysis was carried out using one-way ANOVA (n.s.: non-significant; **p<0.01). [file 12915_2021_965_MOESM2_ESM.tif]

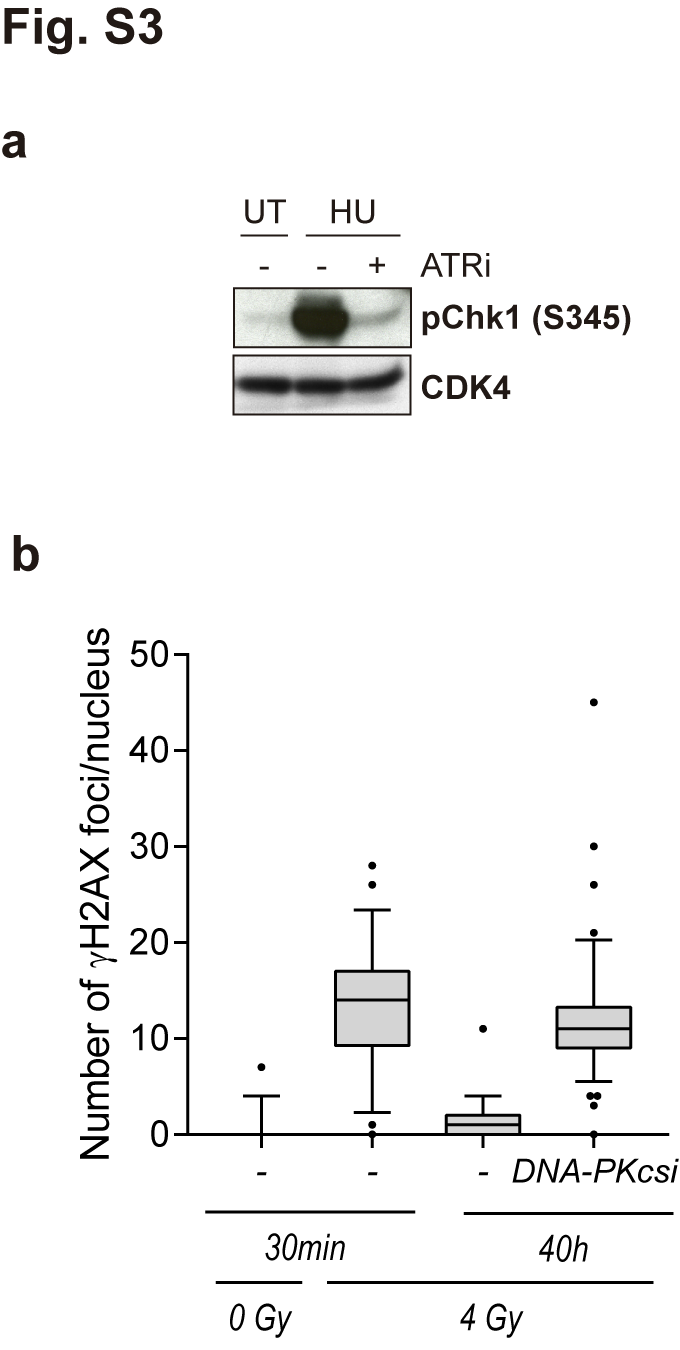

Supplement: Supplementary file 3 — Additional file 3: Fig. S3. Controls for ATR and DNA-PKcs inhibitors used in the present work. a RPE-1 cells were treated with hydroxyurea (HU) to induce ATR activation, or pre-treated with ATR inhibitor before HU treatment. Protein was harvested, and ATR activation status was checked by western blot using pCHK1 S345 phopshosite as a readout. CDK4 served as a loading control. ATRi effectively prevented HU-induced pCHK2 phosphorylation. b G1-synchronized RPE-1 cells grown onto coverslips were irradiated (4 Gy), and fixed for γH2AX and DAPI staining at the indicated timepoints; one sample was pretreated with DNA-PKcs inhibitor. Treatment with DNA-PKcsi prevented the foci resolution observed at 40 hours post-damage. [file 12915_2021_965_MOESM3_ESM.tif]

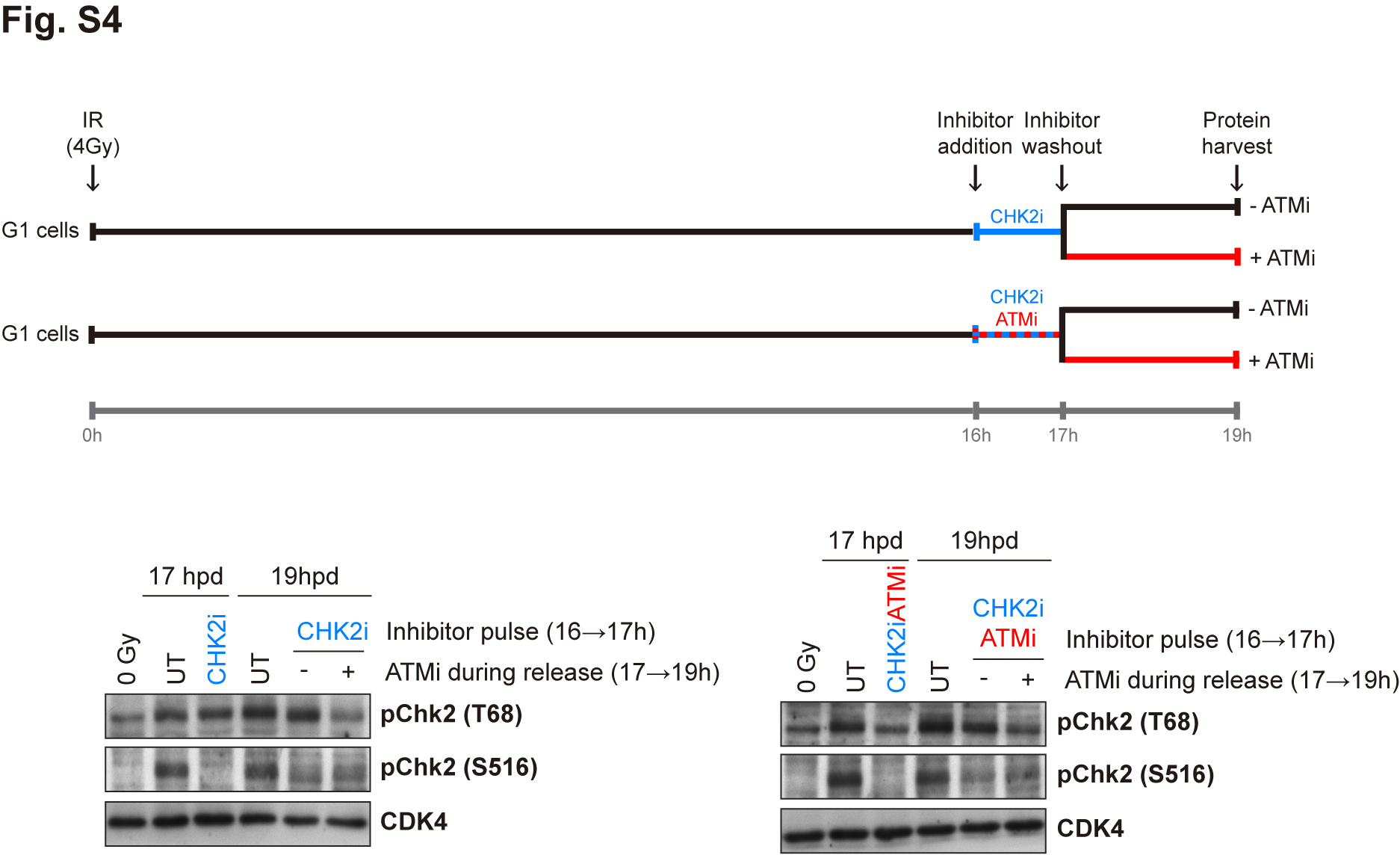

Supplement: Supplementary file 4 — Additional file 4: Fig. S4. CHK2 self-activity at long timepoints after DNA damage enables G1 arrest establishment. Upper panel: experimental setup. G1-synchronized RPE-1 cells were irradiated with a dose of 4 Gy, and 16h after IR inhibitors for either CHK2 alone (CHK2i, blue line) or CHK2 and ATM (blue and red line) were added; one hour later (17h timepoint) inhibitors were washed out, and cells were incubated in the absence (-ATMi, black line) or presence (+ATMi, red line) of ATM inhibitor for two additional hours (19 h timepoint). Lower panel. western blot analysis of protein extracts from 17h and 19h timepoints; left panel corresponds to cells treated with CHK2i alone, and right panel to cells treated with a combination of CHK2i and ATMi. [file 12915_2021_965_MOESM4_ESM.tif]
