# Supplementary material for: Sustained CHK2 activity, but not ATM activity, is critical to maintain a G1 arrest after DNA damage in untransformed cells
Source: BMC Biol. 2021 Feb 19;19:35. doi: 10.1186/s12915-021-00965-x (PMC7896382; doi:10.1186/s12915-021-00965-x)
Supplement: Supplementary file 5 — Additional file 5: Fig. S5. Uncropped western blots. [file 12915_2021_965_MOESM5_ESM.pdf]

Fig. S5 (part 1)

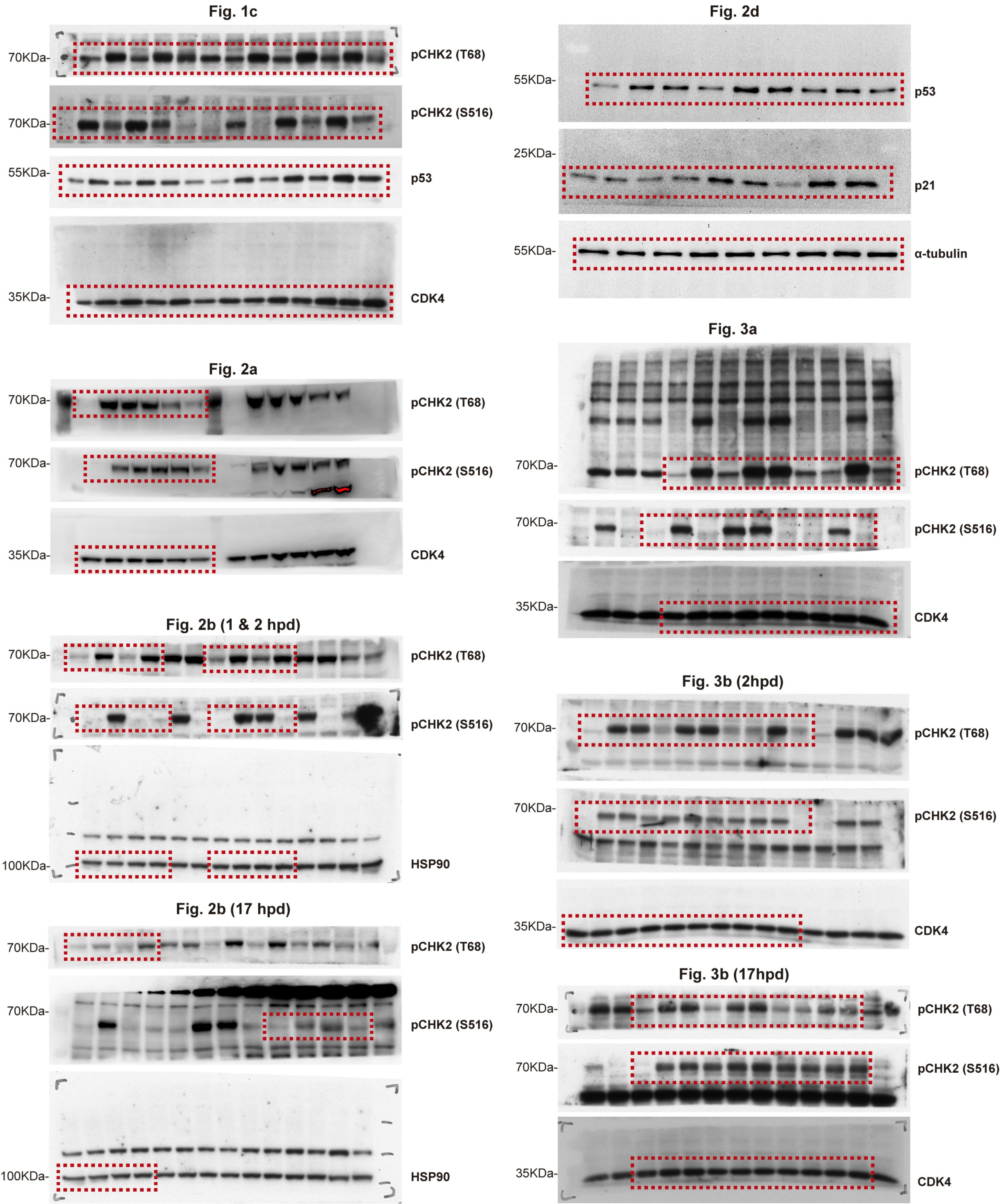

Fig. 1c
70KDa-
pCHK2 (T68)
70KDa-
pCHK2 (S516)
55KDa-
p53
35KDa-
CDK4
Fig. 2a
70KDa-
pCHK2 (T68)
70KDa-
pCHK2 (S516)
35KDa-
CDK4
Fig. 2b (1 & 2 hpd)
70KDa-
pCHK2 (T68)
70KDa-
pCHK2 (S516)
100KDa-
HSP90
Fig. 2b (17 hpd)
70KDa-
pCHK2 (T68)
70KDa-
pCHK2 (S516)
100KDa-
HSP90
Fig. 2d
55KDa-
p53
25KDa-
p21
55KDa-
α-tubulin
Fig. 3a
70KDa-
pCHK2 (T68)
70KDa-
pCHK2 (S516)
35KDa-
CDK4
Fig. 3b (2hpd)
70KDa-
pCHK2 (T68)
70KDa-
pCHK2 (S516)
35KDa-
CDK4
Fig. 3b (17hpd)
70KDa-
pCHK2 (T68)
70KDa-
pCHK2 (S516)
35KDa-
CDK4

Fig. S5 (part 2)

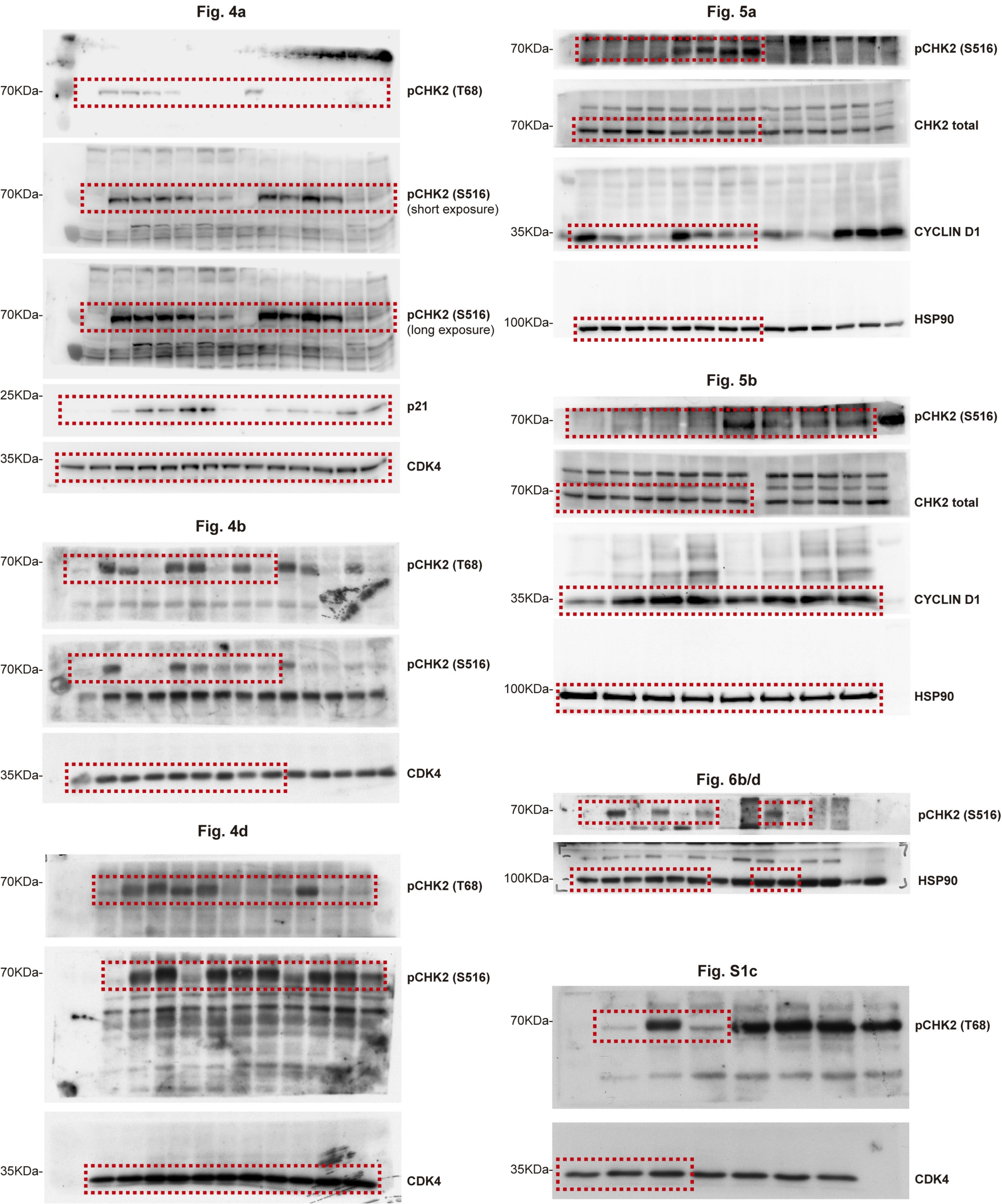
Fig. 4a
70KDa-
pCHK2 (T68)
70KDa-
pCHK2 (S516)
(short exposure)
70KDa-
pCHK2 (S516)
(long exposure)
25KDa-
p21
35KDa-
CDK4
Fig. 4b
70KDa-
pCHK2 (T68)
70KDa-
pCHK2 (S516)
35KDa-
CDK4
Fig. 4d
70KDa-
pCHK2 (T68)
70KDa-
pCHK2 (S516)
35KDa-
CDK4
Fig. 5a
70KDa-
pCHK2 (S516)
70KDa-
CHK2 total
35KDa-
CYCLIN D1
100KDa-
HSP90
Fig. 5b
70KDa-
pCHK2 (S516)
70KDa-
CHK2 total
35KDa-
CYCLIN D1
100KDa-
HSP90
Fig. 6b/d
70KDa-
pCHK2 (S516)
100KDa-
HSP90
Fig. S1c
70KDa-
pCHK2 (T68)
35KDa-
CDK4

Fig. S5 (part 3)

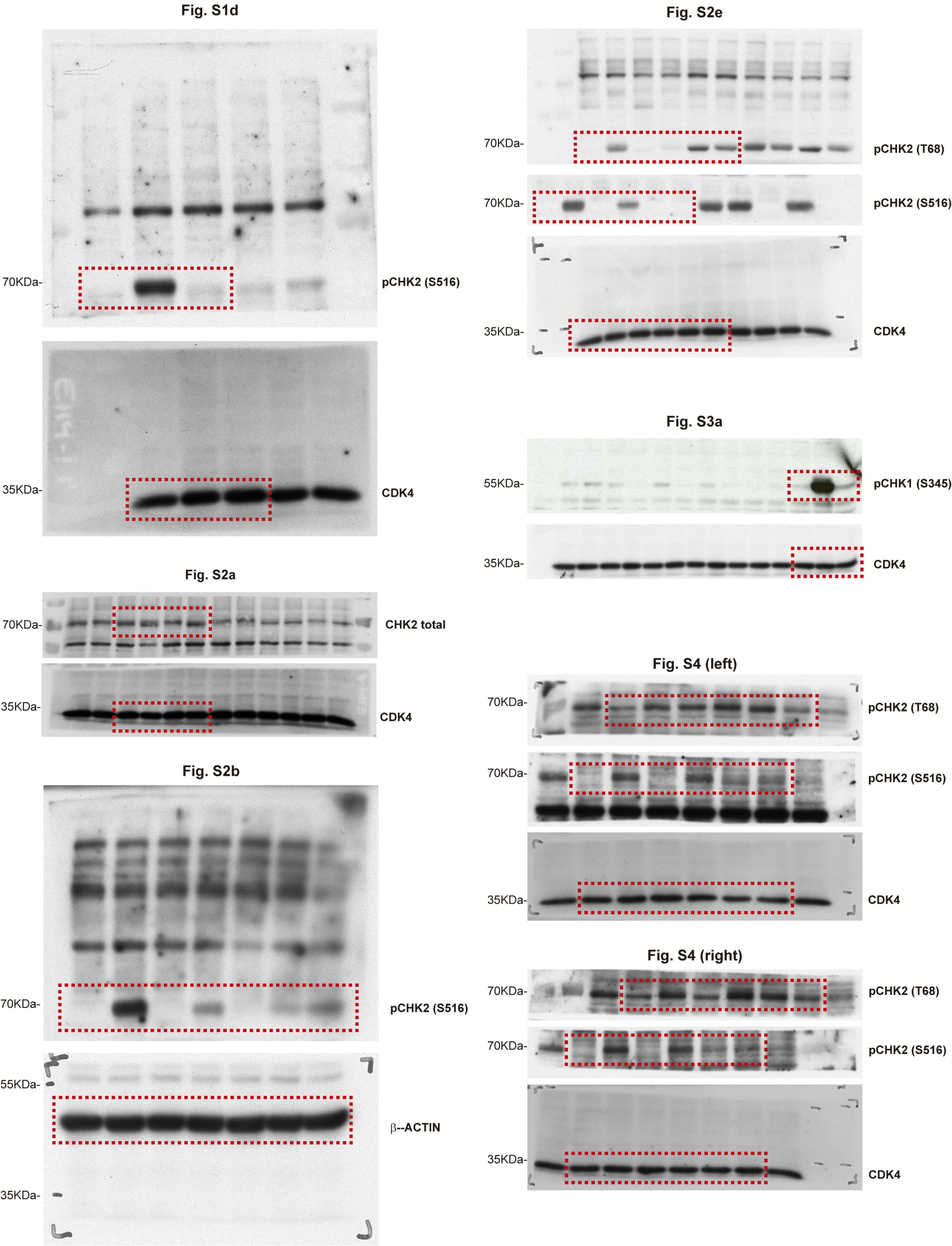

Fig. S1d
70KDa-
pCHK2 (S516)
35KDa-
CDK4
Fig. S2a
70KDa-
CHK2 total
35KDa-
CDK4
Fig. S2b
70KDa-
pCHK2 (S516)
55KDa-
β--ACTIN
35KDa-
Fig. S2e
70KDa-
pCHK2 (T68)
70KDa-
pCHK2 (S516)
35KDa-
CDK4
Fig. S3a
55KDa-
pCHK1 (S345)
35KDa-
CDK4
Fig. S4 (left)
70KDa-
pCHK2 (T68)
70KDa-
pCHK2 (S516)
35KDa-
CDK4
Fig. S4 (right)
70KDa-
pCHK2 (T68)
70KDa-
pCHK2 (S516)
35KDa-
CDK4
